# Accuracy of landmark scalp blocks performed during asleep-awake-asleep awake craniotomy: a retrospective study

**DOI:** 10.1186/s40981-021-00412-4

**Published:** 2021-01-09

**Authors:** Takehito Sato, Kimitoshi Nishiwaki

**Affiliations:** Department of Anesthesiology, Nagoya University hospital, 65 Tsurumai-cho, Showa-ku, Nagoya City, Aichi 466-8550 Japan

To the Editor,

Awake craniotomy (AC) is often performed in patients with brain tumors that are present in regions linked to language processing to minimize any damage to language functioning [1]. During ACs, scalp blocks affecting the supraorbital nerve, supratrochlear nerve, greater and lesser occipital nerve, auriculotemporal nerve, and zygomaticotemporal branch nerve enable safe surgical procedure during the awake phase of ACs [1, 2]. If the scalp block is not effective, it becomes difficult to perform any awake tasks with the cooperation of the patient, as performance is hindered due to headache.

Scalp blocks in ACs are often performed under general anesthesia; however, at our hospital, we perform scalp blocks before the patient is anesthetized to achieve less headache during the awake phase [3]. If the block effect is insufficient as no sensation reduction is demonstrated by the cold test after blocking, an additional dosage of local anesthetic can be administered. To date, no study has considered the accuracy of scalp blocks performed by landmark methods. In this study, we retrospectively investigated the accuracy of scalp blocks performed using the landmark method in ACs.

This study was approved by the Ethics Committee of Nagoya University (Approval number: 2020-0108). We investigated retrospective cases of ACs (*n* = 103) performed at Nagoya University Hospital from January 1, 2016, to May 30, 2020. We performed all scalp blocks bilaterally by landmark methods as described previously with 0.375% ropivacaine [1, 3]: 1.5–2 mL for supraorbital/supratrochlear nerves, 2–2.5 mL for greater/lesser occipital nerves, 3–5 mL for auriculotemporal nerve, and 3–5 mL for zygomaticotemporal branch nerve (total number, 206). After all of the scalp blocks were performed, we assessed the number of block failures on each side by using the cold test before induction. We compared the number of block failures using Cochran’s *Q* test. The statistical analyses were performed with EZR (Saitama Medical Center, Jichi Medical University [4]). All data were analyzed using Cochran’s *Q* test and McNemar test with the post hoc Bonferroni test. *P* < 0.05 was considered statistically significant.

The rates of failure in the scalp blocks were as follows: supraorbital and supratrochlear nerve (*n* = 5, failure rate 2.4%), greater and lesser occipital nerve (*n* = 15, 7.2%), auriculotemporal nerve (*n* = 23, 11.1%), and zygomaticotemporal branch (*n* = 34, 16.6%).

Among these blocks, the zygomaticotemporal nerve block failure rate was significantly higher than in other nerves (*P* < 0.001, at the Cochran’s *Q* test, versus supraorbital and supratrochlear nerve (*P* < 0.001), greater and lesser occipital nerve (*P* < 0.001), auriculotemporal nerve (*P* = 0.015)).

This study revealed the failure rate for zygomaticotemporal nerve blocks was higher than that of the other nerve blocks. The zygomaticotemporal nerve is distributed deep below the surface of the skin and can have anomalies [5, 6]; therefore, it can be difficult to anesthetize using the anatomical landmark method. Patients often suffer headaches during AC, and as the zygomaticotemporal nerve is a nerve responsible for the sensation in the surrounding temple [6], it may be the cause of temporal pain during an AC.

To improve the success rate of the zygomaticotemporal nerve, further studies assessing different blocking techniques, such as using ultrasounds, are required in the future.

## Data Availability

They are available as an Excel® file upon reasonable request.
